# Comparative outcomes of resting splints and elastic bandages in adult lower extremity soft tissue trauma: A retrospective cohort study

**DOI:** 10.1097/MD.0000000000048697

**Published:** 2026-05-08

**Authors:** Volkan Özel, İbrahim Halil Demir, Kazim Ersin Altinsoy

**Affiliations:** a Sait Ertürk Etimesgut State Hospital, Orthopedics and Traumatology Clinic, Ankara, Turkey; b Department of Orthopaedics and Traumatology, Gaziantep City Hospital Hospital, Gaziantep, Turkey; c Department of Emergency medicine, Gaziantep City Hospital, Gaziantep, Turkey.

**Keywords:** compression bandages, injury, lower extremity, pain management, splints

## Abstract

To compare the clinical effectiveness and patient compliance of resting splints and elastic bandages in the management of adult lower extremity soft tissue injuries without fractures. This retrospective cohort study included 659 adult patients (≥18 years) presenting with lower extremity trauma between September 2023 and September 2024; only patients without fractures who completed at least 8 weeks of follow-up were included. Patients were treated either with a splint (n = 392) or elastic bandage (n = 267). Pain was assessed using the visual analog scale at admission, post-immobilization, and follow-up. Early removal rates and patient compliance were also evaluated. Mean visual analog scale scores at admission were higher in the splint group (8.85) compared to the elastic bandage group (7.77, *P* < .001). Both groups showed significant pain reduction at follow-up, with no significant difference between them (*P* = .078). The early removal rate was significantly higher in the splint group (39.8% vs 15.7%, *P* < .001). Splints were more frequently removed due to tightness (12.2%). No splint type, application site, or presence of open wounds significantly affected early removal. Elastic bandages provide comparable pain relief and better patient compliance compared to splints in lower extremity soft tissue trauma. Given their lower cost and fewer complications, elastic bandages may be a preferred alternative in selected cases.

## 1. Introduction

Trauma is a condition that affects all age groups and is considered a significant public health issue due to its high treatment costs and the associated loss of workforce. Lower extremity injuries are among the most frequently encountered types of injuries in trauma patients.^[1]^ Splinting has long been a fundamental approach in managing lower extremity musculoskeletal injuries across various clinical and emergency settings. However, splinting has not significantly benefited from notable medical advancements in terms of technique or material diversity over time.^[2]^

Effective splinting of an acutely injured soft tissue extremity has been well-documented to improve pain control, reduce blood loss, and contribute to the protection of soft tissues. The effectiveness of splinting is directly related to the quality of the materials used and the skill level of the practitioner.^[3,4]^ A properly applied splint should adequately stabilize and protect bone structures in the desired position while minimizing the risk of damage to the surrounding soft tissues. Additionally, splint application must be reproducible, as it is often performed by individuals with varying levels of medical knowledge and experience.^[2]^

For this reason, incorrect applications of resting splints are frequently encountered. Improperly applied or excessively prolonged splint use can lead to severe complications such as wounds, necrosis, compartment syndrome, and reflex sympathetic dystrophy in the affected extremity.^[5]^ To avoid these complications, elastic bandages, which are more practical, objective, and cost-effective, have become an alternative in recent years.^[6]^ However, no studies in the literature have directly compared these 2 treatment modalities.

The aim of this retrospective cohort study was to describe the epidemiological and clinical characteristics of adult patients presenting with lower extremity soft-tissue trauma and to compare resting splint versus elastic bandage immobilization in terms of pain trajectory (visual analog scale [VAS] at presentation, post-immobilization, and follow-up), patient compliance (premature removal rates and reasons), and factors associated with early removal.

## 2. Materials and methods

### 2.1. Ethical approval

After obtaining informed consent from the participants, the study was carried out in accordance with the principles of the Declaration of Helsinki and was approved by the relevant Non-Interventional Clinical Research Ethics Committee (Approval No: 2025-2ÖNP-0009).

The data for this retrospectively designed study were obtained from patients aged 18 years and older who presented to the emergency department and orthopedic outpatient clinic with lower extremity trauma between September 1, 2023, and September 1, 2024, at 2 centers, and who had a minimum follow-up period of 8 weeks. Study data were obtained by searching for lower extremity trauma-related diagnosis codes in the Hospital Document Management System using the International Classification of Diseases, Tenth Revision (Table 1).

**Table 1:** ICD-10 diagnosis codes screened in the study for lower extremity soft tissue injuries.

ICD-10 code	Description
S90	Superficial injury of the ankle and foot
S93	Dislocation, sprain, and strain of joints and ligaments at the ankle and foot level
S96	Injury of muscle and tendon at the ankle and foot level
S99	Other and unspecified injuries of the ankle and foot
S83	Dislocation, sprain, and strain of the knee joint and ligaments
S87	Crushing injury of the lower leg
S80	Superficial injury of the lower leg

ICD-10 = International Classification of Diseases, Tenth Revision.

**Figure 1.**
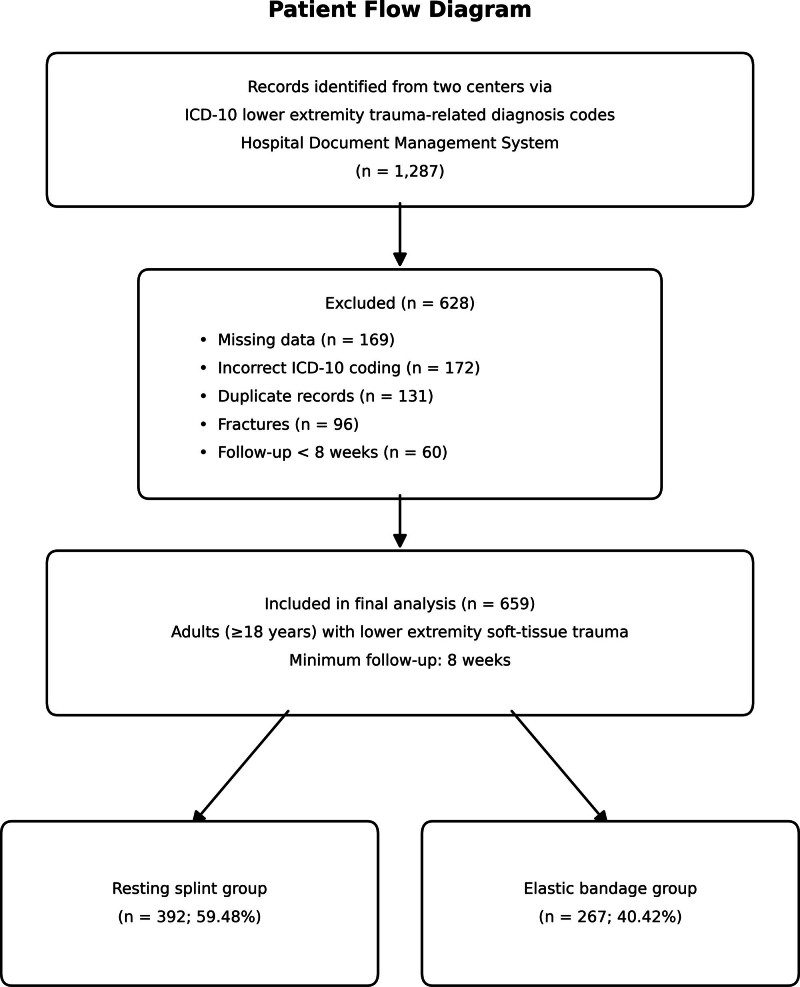
Patient flow diagram for cohort identification, screening, and allocation to treatment groups. Records were retrieved from the hospital document management systems of 2 participating centers using ICD-10 diagnosis codes related to lower extremity trauma (n = 1287). Records were excluded for missing data (n = 169), incorrect ICD-10 coding (n = 172), duplicate entries (n = 131), fractures (n = 96), or follow-up shorter than 8 weeks (n = 60), yielding 659 eligible adults (≥ 18 years) with lower extremity soft-tissue trauma and a minimum follow-up of 8 weeks. The final cohort comprised 392 patients managed with a resting splint (59.48%) and 267 managed with an elastic bandage (40.42%). ICD-10 = International Classification of Diseases, 10th Revision, n = number of patients.

Patients with missing data, incorrect International Classification of Diseases, Tenth Revision coding, duplicate records, fractures, or a follow-up period of less than 8 weeks were excluded from the study (Fig 1). Patients were evaluated based on variables including age, sex, occupation, type of trauma, time of trauma, timing and location of splint or elastic bandage application, treated anatomical region, follow-up time, and VAS scores before, after, and at follow-up. The VAS score was obtained by patient self-report, with patients marking their pain intensity on the VAS. Additionally, patient records were reviewed to determine whether the splint or elastic bandage was removed before follow-up. Immobilization method (resting splint vs elastic bandage) was selected in routine clinical practice rather than randomized, potential confounding was anticipated. Variables that could influence both treatment choice and outcomes (including baseline VAS score [as a proxy for symptom severity], anatomical region [knee/ankle/foot], trauma mechanism, presence of an open wound, and place of application [emergency department vs outpatient clinic]) were collected and considered in the analyses. Where applicable, adjusted models were used to control for these factors, and residual confounding due to unmeasured injury severity or clinician-related factors may persist.

### 2.2. Power analysis (two independent groups)

#### 2.2.1. Assumptions

- Cohen d = 0.54
- n_1_ = 392 (Splint group), n_2_ = 267 (Elastic bandage group)
- α = 0.05
- Test type = 2-tailed independent samples *t*-test
- Statistical Power (1-β) ≈ 99.4%

#### 2.2.2. Conclusion

This study demonstrates:

- A moderate effect size (d ≈ 0.54)
- A large sample size

Therefore, the statistical power to detect a difference in initial VAS scores between the 2 groups is very high, exceeding 99%.

### 2.3. Statistical analysis

The statistical analyses were performed using Statistical Package for the Social Sciences version 27.0 (IBM Corp., Armonk) and MedCalc version 22.007 (MedCalc Software Ltd., Ostend, Belgium). Numerical data were presented as mean ± standard deviation and median (interquartile range), while categorical data were expressed as percentages. The chi-square test was used to compare categorical variables. The distribution of continuous variables was assessed using the Kolmogorov-Smirnov test. For data comparisons, the paired sample *t*-test and independent sample *t*-test were used. All hypotheses were tested bidirectionally, and a *P* value < .05 was considered statistically significant. To evaluate temporal changes in pain within each group, VAS scores at baseline, post-immobilization, and follow-up were compared using paired samples *t*-tests. Additionally, change scores (ΔVAS) were calculated as baseline minus the subsequent measurement (baseline→post-immobilization and baseline→follow-up), with positive values indicating pain reduction; between-group differences in ΔVAS were assessed using Welch *t*-test.

Because treatment allocation was non-randomized, adjusted analyses were performed to control for baseline differences in pain and other potential confounders. Analysis of covariance-style multivariable linear regression models were fitted for post-immobilization VAS and follow-up VAS, including treatment group (elastic bandage vs splint) as the primary predictor and adjusting for baseline VAS, age, sex, trauma mechanism, treated anatomical region, open wound status, and place of application; follow-up interval (days) was additionally included in the follow-up VAS model. Premature removal of immobilization (yes/no) was examined using multivariable logistic regression with the same covariates (including follow-up interval). Robust (HC3) standard errors were used for regression models. All tests were 2-sided, and *P* < .05 was considered statistically significant.

## 3. Results

A total of 659 patients were included in the study. Among these, 392 patients (59.48%) underwent splint application following lower extremity trauma, while 267 patients (40.42%) received elastic bandage treatment.

The mean age of patients who received splint application was 37.28 ± 19.14 years, whereas the mean age of those treated with an elastic bandage was 37.45 ± 19.78 years. In the study group, 51.3% of patients in the splint group and 53.9% in the elastic bandage group were male. Falls were identified as the most common trauma mechanism in both groups (Table 2).

**Table 2:** Demographic and clinical characteristics of the study participants.

Variable	Splint (n = 392)	Elastic bandage (n = 267)
Age	37.28 (19.14)	37.45 (19.78)
Gender		
Male	201 (51.3%)	144 (53.9%)
Female	191 (48.7%)	123 (46.1%)
Trauma Mechanism		
Fall	311 (79.3%)	164 (61.4%)
Assault	39 (9.2%)	54 (20.2%)
Traffic Accident	42 (10.7%)	49 (18.4%)
Occupation		
Worker	68 (17.3%)	40 (14.9%)
Officer	55 (14.2%)	26 (9.8%)
Student	116 (29.5%)	75 (28.1%)
Housewife	76 (19.4%)	53 (19.8%)
Soldier	33 (8.4%)	36 (13.5%)
Tradesman	44 (11.2%)	37 (13.9%)
Immobilized Region		
Thumb	19 (4.8%)	na
Fifth Toe	149 (38%)	na
Foot	149 (38%)	72 (27%)
Ankle	32 (8.2%)	95 (35.6%)
Knee	14 (3.6%)	66 (24.7%)
Leg	29 (7.4%)	34 (14.7%)
Presence of Open Wound in Application Area		
No	355 (90.6%)	203 (76%)
Yes	37 (9.4%)	64 (24%)
Location of Immobilization		
Emergency Department	360 (91.8%)	183 (68.5%)
Orthopedic Outpatient Clinic	32 (8.2%)	84 (31.5%)
Type of Splint		
Short Leg Splint	332 (84.7%)	na
Long Leg Splint	60 (15.3%)	na

Values are presented as mean (SD) for continuous variables and n (%) for categorical variables.

n = number of patients, na = not applicable, SD = standard deviation.

In 91.8% of the patients who received splint treatment, the procedure was performed in the emergency department (Table 2). Among all study patients, 101 had open wounds, and 63.3% of these patients were treated with elastic bandage application.

### 3.1. Patient compliance with immobilization

Among the patients who were immobilized with a splint, 39.8% removed the splint before their scheduled follow-up appointment. In contrast, this rate was significantly lower in the group treated with an elastic bandage, with 15.7% of patients removing it prematurely. The most commonly reported reason for early removal was the resolution of pain.

### 3.2. Reasons for early removal and discharge summary

In the splint group, 12.2% of patients reported removing the splint due to it being too tight. No patients in the elastic bandage group had complaints of tightness (Table 2).

After the follow-up examination, 48.4% of patients in the splint group were discharged without any additional treatment recommendations. 41.7% of these patients were advised to continue with elastic bandage treatment, while 9.8% were prescribed a brace.

In contrast, all patients in the elastic bandage group were discharged without any further recommendations (Table 3).

**Table 3:** Treatment-related characteristics of study patients.

Variable	Splint (n = 392)	Elastic bandage (n = 267)
Early removal of immobilization		
Yes	156 (39.8%)	42 (15.7%)
No	236 (60.2%)	225 (84.3%)
Reason for early removal		
No	235 (59.9%)	225 (84.3%)
Pain relief	87 (22.2%)	37 (13.9%)
Itching	22 (5.6%)	58 (1.9%)
Tightness	48 (12.2%)	na
Desire to remove after follow-up		
Yes	180 (45.7%)	197 (73.8%)
No	212 (54.1%)	70 (26.2%)
Medication recommended after removal		
None	187 (48.4%)	267 (100%)
Elastic bandage	161 (41.7%)	na
Brace	38 (9.8%)	na

Values are presented as n (%).

n = number of patients, na = not applicable.

The splint group had a significantly higher pain intensity at presentation compared with the elastic bandage group (VAS: 8.85 ± 1.15 vs 7.78 ± 1.50; *P* < .001). No significant between-group difference was observed in post-immobilization measurements (VAS: 4.29 ± 1.93 vs 4.24 ± 2.40; *P* = .788). Similarly, VAS scores assessed at outpatient follow-up were comparable between the 2 groups (VAS: 2.18 ± 2.37 vs 2.05 ± 2.46; *P* = .496).

When pain reduction relative to baseline (ΔVAS) was evaluated, the splint group demonstrated a greater decrease from presentation to post-immobilization than the elastic bandage group (ΔVAS: 4.56 ± 2.10 vs 3.53 ± 2.20; *P* < .001). Total pain reduction from presentation to follow-up was also higher in the splint group (ΔVAS: 6.67 ± 2.56 vs 5.72 ± 2.61; *P* < .001). However, in adjusted analyses controlling for between-group differences in baseline pain and potential confounders, no significant difference was found between groups for post-immobilization VAS (*β* = 0.36, 95% confidence interval [CI] [−0.03, 0.75], *P* = .072) or follow-up VAS (*β* = −0.08, 95% CI [−0.43, 0.28], *P* = .673). These findings suggest that the unadjusted ΔVAS differences may largely reflect baseline pain imbalances, and that both immobilization methods achieved similar pain levels after adjustment (Table 4).

**Table 4:** Temporal changes in VAS scores (0–10) and between-group comparisons (unadjusted and adjusted).

Time point/ parameter	Splint (n = 392) M (SD)	Elastic bandage (n = 267) M (SD)	Unadjusted *P*	Adjusted difference (*β*) [95% CI]	Adjusted *P*
Initial presentation (baseline)	8.85 (1.15)	7.78 (1.50)	< .001	na	na
Post-immobilization	4.29 (1.93)	4.24 (2.40)	.788	0.36 [−0.03, 0.75]	.072
Outpatient follow-up	2.18 (2.37)	2.05 (2.46)	.496	−0.08 [−0.43, 0.28]	.673
Change (Δ) baseline → post-immobilization	4.56 (2.10)	3.53 (2.20)	< .001	na	na
Change (Δ) baseline → follow-up	6.67 (2.56)	5.72 (2.61)	< .001	na	na

VAS (0–10; higher scores indicate greater pain). Values are presented as mean (SD). Unadjusted *P* values are from Welch independent-samples *t*-tests. Adjusted differences (*β*) compare elastic bandage with splint and are from ANCOVA-style multivariable linear regression models controlling for baseline VAS, age, sex, trauma mechanism, treated region, open wound status, and place of application; follow-up interval (days) was additionally included in the follow-up VAS model. Robust (HC3) standard errors were used. Δ values were calculated as baseline minus the later time point; positive values indicate pain reduction.

ANCOVA = analysis of covariance, M = mean, n = number of patients, na = not applicable, SD = standard deviation, VAS = visual analog scale.

When the factors influencing the early removal of splints before the control day in the splint group were examined, no significant relationship was found between the presence of open wounds, the type of splint, and the immobilized area (table 5).

**Table 5:** Factors associated with premature removal of the resting splint.

Variable	Early removal: Yes, n	Early removal: No, n	Total, n	*P*
Presence of open wound				.290
Yes	18	19	37	
No	138	217	355	
Type of splint				.349
Long leg splint	22	38	60	
Short leg splint	134	198	332	
Place of splint application				.271
Emergency department	31	152	183	
Outpatient clinic	11	73	84	

*P* values are from chi-square tests. Values are presented as counts (n). For the “place of splint application” block, the provided totals sum to 267 rather than 392; please verify these counts in the source dataset.

n = number of patients.

When examining the factors affecting the early removal of the elastic bandage, it was found that the location of the elastic bandage was significantly associated with early removal (*P* = .015). Specifically, the elastic bandage applied to the knee area was statistically significantly removed earlier (Table 6).

**Table 6:** Factors associated with premature removal of the elastic bandage.

Variable	Early removal: Yes, n	Early removal: No, n	Total, n	*P*
Presence of open wound				.156
Yes	35	168	203	
No	7	57	64	
Location of elastic bandage				.015
Foot	7	65	72	
Ankle	10	85	95	
Place of application				.271
Emergency department	31	152	183	
Outpatient clinic	11	73	84	

*P* values are from chi-square tests. Values are presented as counts (n).

n = number of patients.

The counts for “location of elastic bandage” sum to 167 rather than 267; please verify whether additional locations/categories were present or whether cases were missing for this variable.

The relationship between the early removal of the immobilization method and the calculated VAS scores was examined. In the splint group, it was found that the group who removed the splint themselves had significantly lower VAS scores during the follow-up examination (*P* < .001). In the elastic bandage group, unlike the splint group, it was observed that the group with a decrease in VAS scores after the immobilization procedure also had the elastic bandage removed earlier more frequently (*P* = .017) (Table 7).

**Table 7:** Relationship between premature removal of immobilization and VAS scores.

Outcome	Splint group			Elastic bandage group		
	Early removal: Yes, M	Early removal: No, M	p	Early removal: Yes, M	Early removal: No, M	*P*
VAS score at admission	8.88	8.83	.890	7.31	7.85	.518
VAS score after immobilization	4.15	4.38	.110	4.12	4.27	.017
VAS score at follow-up	1.60	2.57	< .001	1.88	2.27	< .001

VAS (0–10; higher scores indicate greater pain). Values are presented as means (M). *P* values are from independent-samples *t*-tests comparing patients who removed the immobilization early versus those who did not, within each treatment group.

M = mean, VAS = visual analog scale.

In the multivariable logistic regression analysis of premature removal (yes/no), adjusted for baseline VAS score, age, sex, trauma mechanism, anatomical region, presence of an open wound, place of application, and follow-up interval, patients treated with an elastic bandage had significantly lower odds of premature removal compared with those treated with a splint (adjusted odds ratio [aOR = 0.23, 95% CI [0.15, 0.37], *P* < .001). This finding suggests that, even after controlling for potential confounders, elastic bandage immobilization was associated with better patient adherence (i.e., a lower likelihood of early removal) than splint immobilization (Table 8).

**Table 8:** Adjusted association between immobilization method and premature removal.

Predictor	Adjusted OR	95% CI	*P*
Elastic bandage vs splint	0.23	[0.15, 0.37]	< .001

Premature removal was modeled with multivariable logistic regression. The model controlled for baseline VAS, age, sex, trauma mechanism, treated region, open wound status, place of application, and follow-up interval (days). Robust (HC3) standard errors were used.

CI = confidence interval, OR = odds ratio, VAS = visual analog scale.

## 4. Discussion

Lower extremity injuries are the most common musculoskeletal injuries seen in emergency departments and outpatient clinics, and they have significant socio-economic impacts.^[7]^ A review of the literature shows that studies are primarily focused on foot and ankle sprains as well as fractures.^[8–11]^ In our study, rather than focusing on a specific trauma mechanism or injury type, all patients with lower extremity soft tissue injuries who presented to the emergency department and outpatient clinics were included, allowing for more generalized results regarding the clinical characteristics of the patients.

In this retrospective study, the mean age of the patients who received splinting was 37.2 years, while the mean age of those who received elastic bandaging was 37.4 years. In the study group, 51.3% of patients in the splint group and 53.9% of patients in the elastic bandage group were male. Falling was the most common trauma mechanism in both groups. A previous study in our country found that the mean age of patients with foot and ankle injuries was 37.2 years, 51.1% of the patients were female, and falling was the most common trauma mechanism.^[12]^ In a meta-analysis by Doherty et al, it was found that ankle sprains were more prevalent in women.^[13]^ In our study, however, lower extremity soft tissue injuries were more common in men, and the mean age was consistent with existing studies.

In our study, no significant difference was found in VAS scores measured during outpatient follow-up between patients who received splints and those who received elastic bandaging for lower extremity injuries without fractures. By the end of the treatment process, both immobilization methods led to similar reductions in VAS scores. There are no studies in the literature comparing these 2 methods for lower extremity injuries without fractures. In a randomized controlled trial conducted by Kearney et al in 2021, splints and removable braces were compared in adults with ankle fractures, and no statistically significant difference was found in terms of ankle function.^[14]^ Additionally, the largest randomized controlled trial (n = 247) showed that removable braces did not perform worse than casts according to the Olerud Molander Ankle Score at 6, 12, and 52 weeks.^[15]^ A study by Demir et al in 2024 also demonstrated that crutches were more effective than casts and walking boots in treating traumatic talus bone marrow edema.^[16]^ Consistent with previous studies, we found no clinically significant difference at any time point between traditional rest splints and elastic bandage treatment in our study.

In a study conducted in our country in 2022, examining the characteristics and treatment costs of patients presenting to the emergency department with foot and ankle injuries, it was found that the average cost of a splint was 102.12 TL, while the cost of an elastic bandage was 34.01 TL.^[17]^ The cost of the splint is nearly 3 times that of the elastic bandage. Furthermore, the application of splints varies depending on the individual and experience, and as a result, splints are often not applied in the optimal position. This leads to patients prematurely removing their splints.^[18]^ In our study, 39.8% of patients in the splint group removed their splints before the scheduled control day, whereas the rate was 15.7% in the elastic bandage group. In addition, in the multivariable logistic regression analysis adjusted for baseline VAS score, age, sex, trauma mechanism, anatomical region, presence of an open wound, place of application, and follow-up interval, elastic bandage application was shown to significantly reduce the likelihood of premature removal compared with splinting (adjusted odds ratio = 0.23; 95% CI, 0.15–0.37; *P* < .001). This finding suggests that, even after controlling for potential confounders, elastic bandage immobilization is associated with better patient adherence (i.e., a lower risk of early removal) than splint immobilization

The literature indicates that patient-specific splints can be manufactured using 3-dimensional (3D) scanning and 3D printing technologies. In a case demonstration, an upper extremity was modeled with a 3D scanner, a 3-mm-thick design was created using computer-aided design, and the splint was printed with polylactic acid, with a reported production time of approximately 66 hours. Although this approach may be clinically advantageous by enabling personalized immobilization without direct contact with the wound area, the prolonged printing time has been emphasized as an important limitation.^[19]^

Similarly, a randomized controlled pilot feasibility trial reported that, in cases requiring at least 4 weeks of immobilization, custom-made splints based on 3D surface scanning were feasible, and patient satisfaction/comfort was comparable to that of thermoplastic splints. Notably, the design process incorporated a 1-mm “offset” to improve comfort and targeted a 3-mm thickness (comparable to thermoplastic materials) using polylactic acid. However, time loss related to post-processing and issues such as material stability/breakage in some cases were reported, and further optimization of the workflow and studies with larger samples were recommended.^[20]^

Taken together, considering the higher premature removal rates observed in the splint group in our study, these findings suggest that future research should evaluate the potential contribution of 3D technology-assisted personalized splints to adherence and comfort in lower extremity injuries, along with their clinical and cost-effectiveness implications

### 4.1. Limitations of the study

The limitations of our study include the retrospective design, which resulted in a high number of patients being excluded due to missing data, the exclusion of pediatric patients from the study, the patient population not being representative of the entire population, and the short follow-up period.

## 5. Conclusion

In adults with lower extremity injuries without fractures, elastic bandaging can be considered an alternative treatment option to traditional rest splinting due to its lower cost.

## Author contributions

**Conceptualization:** Volkan Özel.

**Data curation:** Volkan Özel, İbrahim Halil Demir, Kazim Ersin Altinsoy.

**Formal analysis:** Volkan Özel, İbrahim Halil Demir.

**Funding acquisition:** İbrahim Halil Demir.

**Investigation:** Kazim Ersin Altinsoy.

**Methodology:** Kazim Ersin Altinsoy.

**Writing – original draft:** Volkan Özel.
